# Comparison of screening indicators for different types of thalassemia carriers in Hunan Province

**DOI:** 10.5937/jomb0-46085

**Published:** 2024-04-23

**Authors:** Hua Tang, Rong Yu, Ziyin Yu, Hui Xi

**Affiliations:** 1 Maternal and Child Health Hospital of Hunan Province, Department of Medical Genetic, Changsha City, Hunan Province, China; 2 University of South China, The Affiliated Changsha Central Hospital, Hengyang Medical School, Department of Laboratory, Changsha City, Hunan Province, China

**Keywords:** complete blood cell count, hemoglobin A2, athalassemia, b-thalassemia carrier, thalassemia screening, hemoglobin A2, a-talasemija, nosilac b-talasemije, skrining talasemije

## Abstract

**Background:**

Carrier screening is the most effective method to block the occurrence of thalassemia. However, due to differences in race and genotype, MCV, MCH, HbA2 and other indicators are far from each other. The purpose of this study is to evaluate the common screening indicators of a, b and ab-compound thalassemia carriers in Hunan Province, and try to use the relevant formulas in the existing literature to predict and distinguish different types of thalassemia carriers.

**Methods:**

Receiver operating characteristic curve (ROC curve) combined with Youden index was utilized to analyze results of blood routine examination, hemoglobin electrophoresis, and literature-related formulas for 1111 a-thalassemia carriers, 464 b-thalassemia carriers and 24 ab-thalassemia carriers.

## Abstract (sr)

**Uvod:**

Skrining nosioca je najefikasniji metod za blokiranje pojave talasemije. Međutim, zbog razlika u rasi i genotipu, MCV, MCH, HBA2 i drugi indikatori su daleko jedni od drugih. Svrha ove studije je da proceni uobičajene indikatore skrininga za nosioce talasemije a, b i ab-jedinjenja u provinciji Hunan, i pokuša da koristi relevantne formule u postojećoj literaturi za predviđanje i razlikovanje različitih tipova nosilaca talasemije.

**Metode:**

Kriva operativne karakteristike prijemnika (ROC kriva) u kombinaciji sa Youden-ovim indeksom korišćena je za analizu rezultata rutinskog pregleda krvi, elektroforeze hemoglobina i formula u vezi sa literaturom za 1111 nosioca a-talasemije, 464 nosioca b-talasemije i 24 nosioca thalasemije ab.

## Introduction

Thalassemia is one of the most common hemoglobin disorders [1] [2]. It is estimated that approximately 5% of the population worldwide has at least one variant allele of thalassemia, and as many as 900,000 people are expected to develop clinically significant disease in the early 2000s, most of them in southern China, India, and Southeast Asia [3] [4]. Thalassemias are classified into two main types, α-thalassemia and β-thalassemia. In Fujian, the incidence of α-thalassemia is higher than that of β-thalassemia, at 3.17% [5]. In another study of ours, the carrier rate of thalassemia in Hunan province was 7.10% and 0.12% for αβ thalassemia; the incidence rate of α-thalassemia was 4.83% and that of β-thalassemia was 2.15%. Efficient identification of carriers is the most effective way to prevent thalassemia. Undoubtedly, genetic testing is one of the most accurate methods, but its promotion has certain limitations in less developed areas. Routine blood testing is a popular and low-cost method to obtain many parameters. Since 1970, there have been studies using formulas designed with different blood routine parameters to determine whether it is β-thalassemia minor [6].

Here, we found 1111 α-thalassemia carriers, 464 β-thalassemia carriers, and 24 αβ-thalassemia carriers through blood routine testing, hemoglobin electrophoresis and genetic testing of 12,973 couples from Hunan province who were planning to conceive. The screening efficacy of common screening indicators in blood routine and hemoglobin electrophoresis results was analyzed, and the formulas used for the prediction of β-thalassemia minor [6] [7] were reapplied for predicting α, β and αβ-thalassemia.

## Materials and methods

From 2018 to 2021, 1111 cases of α-thalassemia carriers, 464 cases of β-thalassemia minor and 24 cases of αβ-thalassemia were selected. The formula (RDW*RBCX*HGB)/MCV or log10 (MCH* MCHC*RDW/RBC) was evaluated for the discrimination of the two entities (thalassemia trait and iron deficiency anemia) [8].The common screening indicators in blood routine and hemoglobin electrophoresis results were evaluated by receiver operating characteristic curve (ROC curve) and area under the curve (AUC). When AUC is greater than or equal to 0.70, the positive rate and detection rate were analyzed in combination with Youden index, specific positive rate, specific detection rate, and the appropriate cut-off value for the corresponding index was determined. At the same time, 8 formulas with different blood routine parameters (1: MCV/RBC [9]; 2: MCH/RBC [10]; 3: MCV^2*MCH/100 [11]; 4: MCV-10*RBC [12]; 5: MCV-RBC-3*HGB [12]; 6: MCV-RBC-5*HGB [13]; 7: |80-MCV|*|27-MCH| [7]; 8: HGB/RBC) [14] were employed to discriminate carriers of α, β, αβ-thalassemia, respectively. The accuracy of each formula was compared by ROC-AUC.

## Results

**Figure 1:**
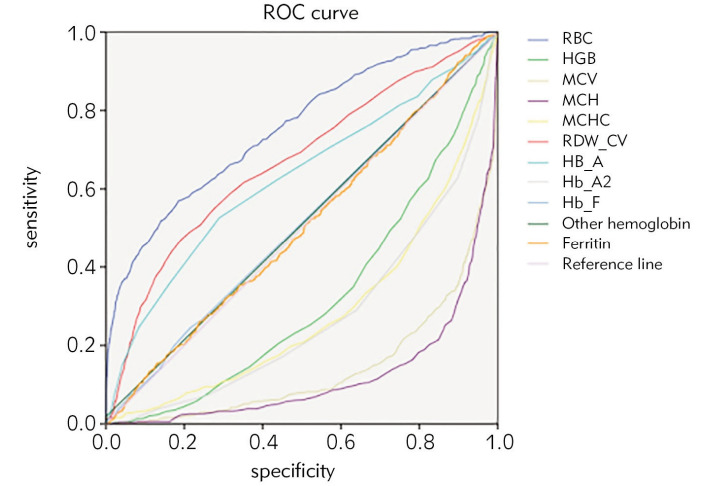
ROC of screening and detection indicators for α-thalassemia carriers.

**Figure 2:**
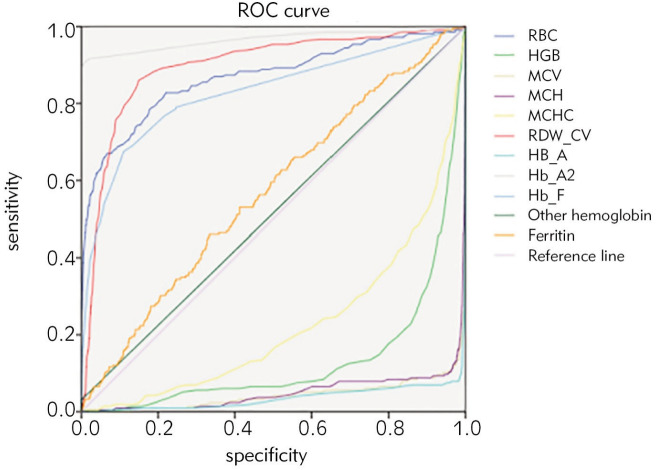
ROC of screening and detection indicators for β-thalassemia minor carriers.

**Figure 3:**
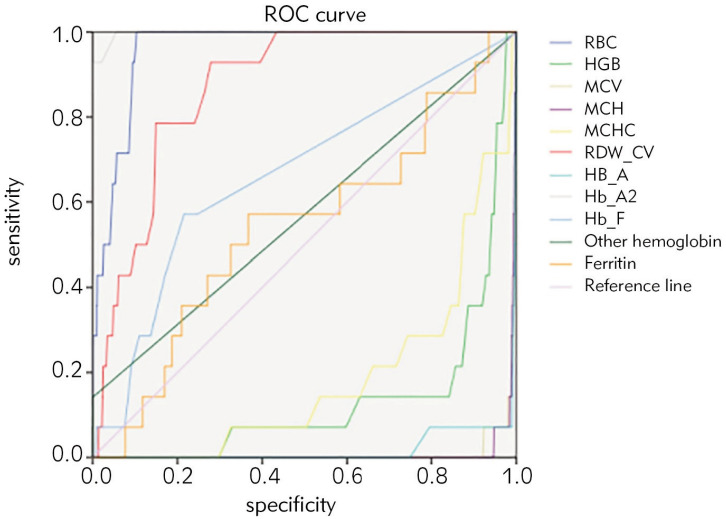
ROC of screening and detection indicators for αβ-thalassemia carriers.

The description of population characteristics and blood parameters are shown in Table 1. ROC curve analysis was performed on the blood routine parameters, hemoglobin electrophoresis indexes and 8 formulas of 1111 cases of α-thalassemia carriers, 464 cases of β-thalassemia minor and 24 cases of αβ-thalassemia (Table 1, Table 2 and Table 3; Figure 1, Figure 2 and Figure 3). According to AUC (0.5-0.7, low efficiency; 0.7-0.9, moderate efficiency; > 0.9, high efficiency), RBC was better for screening α-thalassemia carriers while RBC (AUC 0.868), RDW-CV (AUC 0.896), HbA2 (AUC 0.966), and HbF (AUC 0.828) had better efficiency for screening β-thalassemia minor carriers. At the same time, the formulas MCV-RBC-3*HGB (AUC 0.720), MCV-RBC-5*HGB (AUC 0.760), |80-MCV|*|27-MCH| (AUC 0.707) can be used as screening prediction methods. For screening αβ thalassemia, RBC, RDW-CV, HbA2, HbF were better, and MCV-RBC-3*HGB, MCV-RBC-5*HGB, |80-MCV|*|27-MCH| formulas could be utilized as screening prediction method. The positive rate and detection rate of the indicators were analyzed by Youden index, specific positive rate, and specific detection rate to determine the optimal cut-off value for each indicator.

**Table 1:** ROC analysis of screening and detection indicators for α-thalassemia carriers.

Variables	Area	Standard error^a^	Asymptotic<br>significance^b^	Asymptotic 95% confidence interval	
				Lower limit	Upper limit
RBC	0.756	0.012	0	0.732	0.78
HGB	0.321	0.011	0	0.299	0.343
MCV	0.143	0.009	0	0.125	0.161
MCH	0.122	0.008	0	0.106	0.139
MCHC	0.286	0.012	0	0.262	0.31
RDW-CV	0.669	0.013	0	0.643	0.695
HbA	0.626	0.014	0	0.598	0.654
HbA2	0.275	0.012	0	0.252	0.298
Huff	0.511	0.013	0.403	0.485	0.537
Other hemoglobin	0.509	0.013	0.492	0.483	0.535
MCV/RBC	0.207	0.008	0	0.192	0.223
MCH/RBC	0.185	0.008	0	0.17	0.2
MCV^2*MCH/100	0.127	0.006	0	0.116	0.139
MCV-10*RBC	0.178	0.007	0	0.164	0.192
MCV-RBC-3*HGB	0.578	0.008	0	0.562	0.595
MCV-RBC-5*HGB	0.6	0.008	0	0.583	0.616
|80-MCV|*|27-MCH|	0.284	0.009	0	0.267	0.302
HGB/RBC	0.124	0.006	0	0.112	0.136

**Table 2:** ROC analysis of screening and detection indicators for β-thalassemia carriers.

Variables	Area	Standard error^a^	Asymptotic<br>significance^b^	Asymptotic 95% confidence interval	
				Lower limit	Upper limit
RBC	0.868	0.016	0	0.837	0.899
HGB	0.134	0.014	0	0.107	0.16
MCV	0.044	0.01	0	0.025	0.064
MCH	0.051	0.011	0	0.03	0.072
MCHC	0.225	0.017	0	0.192	0.259
RDW-CV	0.896	0.012	0	0.872	0.919
HbA	0.036	0.009	0	0.018	0.054
HbA2	0.966	0.009	0	0.948	0.985
HbF	0.828	0.018	0	0.793	0.863
Other hemoglobin	0.515	0.02	0.439	0.475	0.555
Ferritin	0.569	0.02	0.001	0.531	0.608
MCV/RBC	0.06	0.008	0	0.044	0.076
MCH/RBC	0.055	0.008	0	0.04	0.071
MCV^2*MCH/100	0.038	0.006	0	0.026	0.05
MCV-10*RBC	0.048	0.007	0	0.034	0.063
MCV-RBC-3*HGB	0.72	0.011	0	0.698	0.742
MCV-RBC-5*HGB	0.76	0.011	0	0.739	0.781
|80-MCV|*|27-MCH|	0.707	0.014	0	0.679	0.735
HGB/RBC	0.038	0.006	0	0.026	0.05

**Table 3:** ROC analysis of screening and detection indicators for αβ-thalassemia carriers.

Variables	Area	Standard error^a^	Asymptotic<br>significance^b^	Asymptotic 95% confidence interval	
				Lower limit	Upper limit
RBC	0.96	0.01	0	0.94	0.98
HGB	0.135	0.047	0	0.042	0.228
MCV	0.011	0.005	0	0	0.021
MCH	0.01	0.003	0	0.004	0.017
MCHC	0.184	0.052	0	0.083	0.285
RDW-CV	0.871	0.03	0	0.812	0.929
HbA	0.02	0.015	0	0	0.051
HbA2	0.997	0.003	0	0.992	1
HbF	0.664	0.078	0.034	0.512	0.816
Other hemoglobin	0.515	0.02	0.439	0.475	0.555
Ferritin	0.569	0.02	0.001	0.531	0.608
MCV/RBC	0.01	0.004	0	0.001	0.019
MCH/RBC	0.005	0.002	0	0.001	0.008
MCV^2*MCH/100	0.006	0.002	0	0.003	0.009
MCV-10*RBC	0.006	0.003	0	0	0.011
MCV-RBC-3*HGB	0.723	0.046	0	0.633	0.813
MCV-RBC-5*HGB	0.76	0.043	0	0.675	0.845
|80-MCV|*|27-MCH|	0.528	0.063	0.637	0.405	0.651
HGB/RBC	0.007	0.001	0	0.005	0.01

The screening efficiency of each type of thalassemia screening index is shown in Table 4. Table 5 and Table 6. It can be seen from Table 4 that even if RBC is used as a screening index for α-thalassemia carriers, no matter which cut-off value is used, the efficiency did not meet expectations. For β-thalassemia minor, RBC (Cut off≥4.58, PR=57.36%, DR=89.01%), RDW-CV (Cut off≥13.85, PR=15.74%, DR=81.68%), HbA2 (Cut off≥3.15, PR=2.89%, DR=81.68%), HBF (Cut off≥0.65, PR=9.04%, DR=56.9%), formula 5 (Cut off≥ 339.49, PR=49.02%, DR=78.88%), formula 6 (Cut off≥ 614.47, PR=45.74%, DR=81.47%) and formula 7 (Cut off≥ 25.12 PR=45.74%, DR=61.85%) can be used. And for the αβ-thalassemia, RBC (Cut off≥4.77, PR=44.51%, DR=100.00%), RDW-CV (Cut off≥13.21, PR=22.55%, DR=83.33%), HbA2 (Cut off≥2.90, PR=17.78%, DR=95.83%), formula 5 (Cut off≥ 335.92, PR=46.38%, DR=83.33%), and formula 6 (Cut off≥ 609.79, PR=43.38%, DR= 83.33%) were suitable. Moreover, HbA2 had high efficiency in the screening of β-thalassemia and αβ-thalassemia. For the screening of β-thalassemia minor, if the cut-off value of HbA2 is set to 3%, the detection rate of 93.32% can be obtained at the positive rate of 9.6%, and if it is set to 3.15%, the detection rate of the positive rate of 2.89% can also reach 81.68%. For αβ-thalassemia, if the cut-off value of HbA2 is set to 3%, the detection rate of 95.83% can be obtained at the positive rate of 8.08%.

**Table 4:** Screening efficiency of screening indicators for α-thalassemia carriers. PR: false positive rate; DR: detection rate

Thalassemia	Indicator	Cut-off value	PR	DR	Efficiency	PR	DR
α	RBC	≥ 4.62	54.97%	75.70%	PR = 5%(≥ 5.56)	5.15%	27.00%
		> 4.62	54.36%	75.43%	PR = 10%(≥ 5.39)	10.21%	36.81%
		≥ 4.6	56.50%	76.42%	DR = 75%(≥ 4.63)	54.36%	75.43%
		> 4.6	55.51%	76.06%	DR = 95%(≥ 4.125)	84.12%	94.96%

**Table 5:** Screening efficiency of screening indicators for β-thalassemia minor carriers. PR: false positive rate; DR: detection rate

Thalassemia	Indicators	Cut-off value	PR	DR	Efficiency	PR	DR
β	RBC	≥4.58	57.36%	89.01%	PR = 5%(≥ 5.55)	5.19%	48.92%
		>4.58	56.80%	88.79%	PR = 10%(≥ 5.38)	10.31%	59.05%
		≥4.63	54.01%	86.42%	DR = 75%(≥ 5.00)	30.92%	75.22%
		>4.63	53.44%	85.99%	DR = 95%(≥ 4.21)	79.07%	94.83%
	RDW_CV	≥13.85	15.74%	81.68%	PR = 5%(≥ 14.70)	5.30%	62.28%
					PR =10%(≥ 14.00)	11.26%	79.74%
					DR = 75%(≥ 14.2)	8.68%	75.65%
					DR = 95%(≥ 12.7)	45.77%	95.04%
	HbA2	≥3.15	2.89%	81.68%	PR = 5%(≥3.00)	9.60%	93.32%
					PR = 10%(≥2.90)	19.15%	94.40%
					DR = 75%(≥4.80)	1.45%	76.29%
					DR = 95%(≥2.80)	34.43%	96.12%
	HbF	≥0.65	9.04%	56.90%	PR = 5%(≥ 1.00)	5.10%	43.10%
					PR = 10%(≥ 0.60)	11.19%	60.78%
					DR = 75%(≥ 0.00)		
					DR = 95%(≥ 0.00)		
	Formula 5	≥ -339.49	49.02%	78.88%	PR = 5%(≥ -253.81)	5.00%	20.04%
		≥ -339.45	49.00%	78.88%	PR=10%(≥ -272.56)	10.00%	31.25%
					DR=75%(≥ -335.31)	46.53%	75.00%
					DR=95%(≥ -376.83)	71.75%	94.83%
	Formula 6	≥ -614.47	45.74%	81.47%	PR = 5%(≥ -479.97)	5.01%	25.00%
		≥ -614.40	45.72%	81.47%	PR = 10%(≥ -511.95)	10.00%	38.79%
					DR = 75%(≥ -601.01)	41.01%	75.00%
					DR = 95%(≥ -674.67)	67.14%	94.83%
	Formula 7	≥ 68.18	25.15%	61.85%	PR = 5%(≥116.51)	5.00%	22.63%
		≥ 68.28	25.12%	61.85%	PR = 10%(≥96.66)	10.01%	38.15%
					DR = 75%(≥ 44.56)	48.61%	75.00%
					DR = 95%(≥ 3.79)	95.86%	94.83%

**Table 6:** Screening efficiency of screening indicators for αβ-thalassemia carriers.

Thalassemia	Indicators	Cut-off value	PR	DR	Efficiency	PR	DR
αβ	RBC	≥ 4.77	44.51%	100.00%	PR = 5%(≥ 5.51)	5.25%	54.17%
					PR = 10%(≥ 5.36)	10.14%	58.33%
					DR = 75%(≥ 4.95)	33.04%	75.00%
					DR = 95%(≥ 4.79)	42.74%	91.67%
	RDW_CV	≥ 13.21	22.55%	83.33%	PR = 5%(≥ 14.50)	5.32%	50.00%
					PR = 10%(≥ 13.90)	10.39%	70.83%
		≥ 13.26	22.54%	83.33%	DR = 75%(≥ 13.62)	14.16%	75.00%
					DR = 95%(≥ 12.48)	53.62%	91.67%
	HbA2	≥ 2.90	17.78%	95.83%	PR = 5%(≥ 3.00)	8.08%	95.83%
					PR = 10%(≥ 2.90)	17.78%	95.83%
		≥ 2.95	8.08%	95.83%	DR = 75%(≥ 5.00)	0.09%	79.17%
					DR = 95%(≥ 3.14)	1.28%	91.67%
	Formula 5	≥ -335.92	46.38%	83.33%	PR = 5%(≥ -255.20)	5.00%	8.33%
		≥ -335.77	46.29%	83.33%	PR = 10%(≥ -273.73)	10.00%	29.17%
					DR = 75%(≥ -329.02)	42.33%	75.00%
					DR = 95%(≥ -367.29)	64.92%	91.67%
	Formula 6	≥ -609.79	43.38%	83.33%	PR = 5%(≥ -483.09)	5.00%	29.17%
		≥ -609.77	43.38%	83.33%	PR = 10%(≥ -514.36)	10.00%	41.67%
					DR = 75%(≥ -597.65)	39.10%	75.00%
					DR = 95%(≥ -655.29)	59.30%	91.67%

## Discussion

Thalassemia is a common genetic disease with abnormal hemoglobin, which has obvious regional distribution and population specificity in the world. About 5% of the world population carries a variant of the α globin gene [15], and the mutation carrier rate of β-thalassemia in the population of Southeast Asia including the Mediterranean coast, the Middle East, and Southern China is 2-30% [16]. At present, the screening of adult populations in China is mainly carried out in premarital and early prenatal examinations. Suspected heterozygotes are first identified by rapid, accurate, and inexpensive hematology methods, and then their genotype is determined by molecular diagnosis. The mainstream approach is phenotype-based screening techniques, with whole blood cell analysis and hemoglobin electrophoresis being the main screening indicators [17]. The former is used for the diagnosis of microcytic hypochromic anemia, and the latter is for the typing of thalassemia. This study aimed to deal with what is the screening performance of relevant indicators for thalassemia carriers, whether certain indicators and corresponding cutoff values can be found in whole blood cell analysis and hemoglobin electrophoresis, and the functional relationship that can be established between many parameters of whole blood cell analysis and the corresponding calculated value can help the judgment of thalassemia by cut-off value, so as to find the corresponding carriers more quickly and efficiently.

The severity of α-thalassemia phenotype directly correlates with the copy number of a gene [15] [18] [19]. Among the 1111 α-thalassemia carriers included in this study, the most common type was αα/-α3.7 557 (50.13%), followed by αα/--SEA 312 (28.08%), αα/-α4.2108 (9.72%), and other types (12.07%), which was different from the reported distribution of α-thalassemia genotypes in other provinces, with regional and population characteristics. In the clinical guidelines recommended in China, MCV < 80 fl, MCH < 27 pg and HbA2 < 2.5% are the screening criteria. Here, ROC was performed with the above indicators, as well as RBC, HGB, MCHC, RDW-CV, HbA, HbF, other hemoglobins and calculated results from 8 formulas for thalassemia prediction. The results showed that for α-thalassemia carriers, only the AUC of RBC exceeded 0.7, and that of other indexes was less than 0.7. The maximum value of the Youden index of RBC was 0.385, and the corresponding RBC was 4.625. It can be seen from Table 4 that when 4.625 is taken as the cut-off value, the positive rate was 54.36% and the detection rate was 75.43%. Even if the cut-off value is increased to 5.39, the positive rate is 10.21%, and the corresponding detection rate is only 36.81%. RBC is also not a good indicator of α-thalassemia carrier screening.

Among the 488 cases of β-thalassemia minor, 136 cases (27.86%) were IVS-II-654 (C>T) β+, 132 cases (27.05%) were codon 41/42 (-TTCT)β, 65 cases (13.32%) were codon 17 (A>T) beta0, 23 cases (4.71%) were codon 71/72(+A) beta0, 22 cases (4.51%) were -28(A>G) beta+, and other types together accounted for 22.55%. Table 2 and Figure 2 showed that the AUC of indicators RBC, RDW-CV, HbA2, HBF and formula 5-7 were all greater than 0.7. Through the analysis of the screening performance of these seven indicators (Table 5), the AUC of HbA2 is the largest. When the cutoff value was ≥ 3.15, the positive rate was 2.89% and the detection rate was 81.68%. If the cutoff value was reduced to 3.0, the positive rate was 9.6% and the detection rate was 93.32%. HbA2 is a better screening index for β-thalassemia minor. In αβ-thalassemia, the AUC of RBC, RDW-CV, HbA2 and formulas 5 and 6 were all greater than 0.7. The screening efficiency analysis showed that when the cut-off value of HbA2 was set at > 2.95, the positive rate was 8.08%, and the detection rate was 8.08%.

Since 1970, there have been reports of using parameters in the complete blood count to design parameters calculated by different formulas to determine whether the population is a β-thalassemia carrier, and the accuracy of different formulas in related studies varies greatly. At present, there are very few studies on the use of complete blood count-related parameters to design formulas for predicting α-thalassemia carriers. Our study failed to use the commonly used screening parameters to find better screening indicators one by one. We tried to use the formula currently used for the prediction of β-thalassemia carriers in the world for the prediction of α-thalassemia carriers and also failed to find a suitable formula. When predicting β-thalassemia carriers, MCV-RBC-3*HGB, MCV-RBC-5*HGB, |80-MCV|*| 27-MCH| all showed better results, but the corresponding sensitivity and specificity were 78.9%/59.6%, 81.5%/55%, 61.9%/73.6%, respectively, which are different from the sensitivity and specificity of the corresponding formulas in other regions. For the prediction of αβ-thalassemia, the corresponding sensitivity/specificity of MCV-RBC-3*HGB and MCV-RBC-5*HGB were 83.3%/53.7% and 83.3%/56.7%. When drawing and analyzing all the detection indicators of α-thalassemia carriers, β-thalassemia carriers and αβ-thalassemia carriers and the normal population, it was found that most of the indicators overlapped with the normal people. Screening for thalassemia carriers by routine blood tests or hemoglobin electrophoresis still misses some. Our study firstly understood the distribution of various important indicators in routine screening of α, β and αβ thalassemia carriers in Hunan province, which provided a basis for clinical understanding of the relevant detection indicators of common thalassemia carriers in Hunan, and also tried to explore ways to improve performance by exploring screening indicators to determine cut-off value in areas where genetic testing cannot be used as a first-line detection method and to explore ways to improve performance. In β-thalassemia carriers and αβ-thalassemia carriers, the Western literature formula was validated for the Chinese population, and its efficacy was confirmed. However, for α-thalassemia carriers, we should further explore the differences in the characteristics of each parameter, and use mathematical methods to amplify the differences in the parameters themselves, and find a mathematical method that can predict thalassemia carriers including α-thalassemia carriers.

## Dodatak

### Acknowledgments 

Not applicable.

### Funding

1. The National Key Research and Development Program of China (2021YFC1005300).

2. Major Scientific and Technological Projects for collaborative prevention and control of birth defects in Hunan Province (2019SK1010).

### Availability of data and materials

The datasets used and/or analyzed during the present study are available from the corresponding author on reasonable request.

### Conflict of interest statement

All the authors declare that they have no conflict of interest in this work.
